# Therapeutic hypoxia to protect and revive ischemic heart and brain

**DOI:** 10.1016/j.mmr.2026.100064

**Published:** 2026-08-19

**Authors:** Johannes Burtscher, Robert T. Mallet, Lei Xi, Katharina Hüfner, Martin Burtscher

**Affiliations:** a Department of Psychiatry, Psychotherapy, Psychosomatics and Medical Psychology, University Hospital for Psychiatry II, Medical University of Innsbruck, 6020 Innsbruck, Austria; b Department of Physiology and Anatomy, University of North Texas Health Science Center, Fort Worth, TX 76107, USA; c Pauley Heart Center, Department of Internal Medicine, Virginia Commonwealth University, Richmond, Virginia, VA 23298, USA; d Institute of Sport Science, University of Innsbruck, 6020 Innsbruck, Austria

**Keywords:** Intermittent hypoxia conditioning (IHC), Remote ischemic conditioning, Cardiovascular, Cerebrovascular, Prevention, Therapy

## Abstract

Although hypoxia plays a central role in the pathobiology of cardiovascular and cerebrovascular diseases, controlled hypoxic stimuli, such as intermittent hypoxia conditioning (IHC), can trigger beneficial adaptations that counteract those diseases. IHC has been studied as a readily administered, non-pharmacological intervention for almost a century, it has not yet been widely adopted in clinical practice. Growing evidence demonstrates the beneficial preventive and therapeutic effects of IHC on the cardiovascular system, and although less conclusively established, on the brain. Moreover, IHC is highly feasible and raises few safety concerns. A notable limitation, similar to that of remote ischemic conditioning, is the lack of refined IHC protocols for personalized, disease-specific applications in the individual patient. Significant progress has been achieved in addressing this limitation. For example, increasingly sophisticated modern hypoxia equipment permits customization of IHC parameters according to each individual’s responses to and tolerance of hypoxia. Still to be evaluated are the persistence of IHC’s effects and the capacity of IHC booster protocols to renew its benefits. Comparative analyses of the mechanisms and efficacy of IHC and remote ischemic conditioning could aid in defining their respective applications and possible synergistic effects. This perspective article summarizes the historical development of IHC, presents a selection of preclinical and clinical studies exemplifying recent research progress, and discusses strategies to address the lingering concerns limiting clinical IHC applications.

## 1 Background

Hypoxia impedes oxidative metabolism, leading to mitochondrial dysfunction, excessive reactive oxygen species (ROS), and cardiomyocyte and neuronal apoptosis, which may provoke cardiac and cerebrovascular disorders including myocardial infarction, heart failure, and stroke [1], [2], [3], [4]. These detrimental effects of hypoxia are exemplified by chronic intermittent hypoxia caused by obstructive sleep apnea, which has a 40%−80% higher prevalence in patients with hypertension, heart failure, coronary artery disease, pulmonary hypertension, atrial fibrillation, and stroke [5]. Conversely, as a fundamental physiological stimulus, hypoxia can trigger potent adaptive responses through stress preconditioning, which may counteract cardiovascular and cerebrovascular disorders when applied by controlled, intermittent forms of hypoxia [intermittent hypoxia conditioning (IHC)] [6], [7], [8]. Stress preconditioning in general, and IHC in particular, exemplify the principle of hormesis, whereby an organism’s adaptive biological responses to various moderate and temporary stressors (e.g., hypoxia, ischemia, cold, heat, radiation, exercise, or fasting) impart resilience to target tissues [8], [9], [10] by myriad molecular and physiological mechanisms [11]. As hormetic stimuli, hypoxia and remote ischemia may be applied before or after injury or disease symptoms to achieve their preventive and/or therapeutic effects [8], [11], [12], [13]. The use of IHC in clinical settings is increasing, as modern devices for delivering hypoxia (e.g., hypoxicators) can precisely adjust exposure parameters according to an individual’s response to hypoxia [14]. Typically, moderate hypoxia is alternated with normoxia or moderately hyperoxia, either at rest or during exercise [7], [14], [15]. The primary focus here is on hypoxia as a standalone stressor, although the effects of hypoxia combined with exercise are also discussed.

## 2 Terminology

The terminology used in the literature to describe various IHC protocols can be confusing, as different terms and acronyms often are applied to the same protocol, or the same term is used for different protocols. For example, intermittent hypoxia training (IHT) and intermittent hypoxia exposure (IHE) usually refer to intermittent hypoxia at rest (passive exposure), although IHT also may refer to hypoxic exposure during exercise (active exposure) [16]. Originally, the hypoxia cycles were interspersed with normoxic cycles, but a newer approach, termed intermittent hypoxia-hyperoxia training (IHHT) replaces hypoxia-normoxia with hypoxia-hyperoxia cycles [14]. Here, all protocols involving hypoxia-normoxia cycles are termed IHC, and those involving hypoxia-hyperoxia cycles are termed intermittent hypoxia-hyperoxia conditioning (IHHC). Also, whether IHC is applied alone or integrated with other interventions, such as exercise, is specified.

## 3 Historical evidence of beneficial effects of altitude/intermittent hypoxia exposure

### 3.1 Observations from continuous altitude hypoxia exposures

In 1960, the Peruvian physician Hurtado [17] reported a low prevalence of cardiovascular disease (CVD), including hypertension, coronary thrombosis, and myocardial infarction, among people residing at 3370 m, thus continuously exposed to hypobaric hypoxia. In addition, Mortimer *et al.* [18] demonstrated lower mortality from coronary artery disease (CAD) in men living at moderate altitudes (914–2000 m) in New Mexico, US. More recently, Ezzati *et al.* [19] reported that people residing in the US counties above 1500 m altitude had life expectancies 1.2–3.6 years longer for men and 0.5–2.5 years longer for women than their counterparts living within 100 m of sea level, primarily ascribable to the protective effects of hypoxia against CVD. Studies from the European Alps also documented reduced mortality from CAD and stroke in residents at moderate (1000–2500 m) vs. low altitudes (<1000 m) [20], [21]. While moderate altitude residence alone may not impart sufficient hypoxic stimulus to induce cardio- and cerebro-protective adaptations, superimposing transiently increased oxygen demand (e.g., during exercise) or reduced oxygen intake (e.g., during sleep) intensifies hypoxic stimuli [7], [22]. Exemplifying this phenomenon, the risk of sudden cardiac death in male mountain hikers and skiers with a history of CAD was significantly lower if they slept at moderate altitudes before undertaking physical activity at higher altitudes [23].

### 3.2 Observations from intermittent hypoxia exposures

The scientific examination of conditioning by intermittent hypoxia began in the 1930s, especially in the former Soviet Union [24], where hypoxia exposures were imparted by repeated stays in mountainous locations, high-altitude airplane flights, the use of hypobaric chambers, or breathing low-oxygen gas mixtures to induce acclimatization to high-altitudes. The first evidence that hypoxic conditioning can confer neuroprotection emerged in the early 1960s. Dahl *et al.* [25] demonstrated that brief exposure of rats to anoxia before a subsequent prolonged anoxic insult significantly improved survival, an effect associated with preservation of cerebral energy metabolism. Beginning in the 1980s and 1990s, extensive studies of IHC treatments (primarily by breathing low-oxygen gas) in humans demonstrated appreciable benefits in myriad clinical disorders, including bronchial asthma, chronic lung diseases, systemic hypertension, and other cardiovascular diseases, diabetes mellitus, depression, and Parkinson’s disease [24]. In the following years, expanding therapeutic hypoxia research revealed additional potential applications [6], [26], [27], [28], [29], [30]. Nevertheless, concerns about therapeutic application of hypoxia persist, particularly given that chronic intermittent hypoxia, as occurs during obstructive sleep apnea, is known to cause cardiovascular disease, metabolic dysfunction, and cognitive impairment [5], [31]. The pathogenesis of chronic intermittent hypoxia includes oxidative stress, persistent systemic inflammation, and protracted sympathetic hyperactivation [5], [31]. These adverse consequences arise from high-frequency (48–2400 cycles/d), intermittent, severe hypoxia [fraction of inspired O_2_ (F_i_O_2_)=0.02–0.08] protocols modeling severe sleep apnea, but those protocols clearly differ from the beneficial, low-dose IHC protocols described above [26]. Armed with this knowledge, a growing number of preclinical and clinical studies have been initiated, focusing on the therapeutic and preventive effects of IHC on the cardiovascular and cerebrovascular systems.

Numerous vertebrate animal studies suggest IHC affords significant cardio- and cerebro-vascular benefits, such as augmented postischemic recovery of myocardial contractile function and/or reduced infarct size in mice, dogs, and rats [28], [29], [30], and promoted somatic motor recovery following spinal injuries in rats and humans [26]. In mice, intermittent, 5-minute exposures to FiO_2_=0.13, 10 exposures per day over two weeks, promoted angiogenesis without compromising blood-brain barrier integrity [32]. This treatment also alleviated neurological dysfunction, reduced cerebral infarct volume, and improved cerebrovascular microcirculation after distal middle cerebral artery occlusion, whereas chronic hypoxia had no such positive effects [32]. A 20-day program of 5–8 daily cycles alternating 5–10 min hypoxia (FiO_2_≈0.10) and 4 min normoxia decreased infarct size by >95% when coronary artery occlusion-reperfusion was imposed 24 h after IHC completion in dogs [7].

In 2004, a randomized controlled trial (RCT) demonstrated that three weeks of passive IHC (FiO₂=0.10–0.14, 3–5 d exposures, each 3–5 min) increased aerobic capacity and exercise tolerance in older men with and without CAD [33]. Subsequent studies have corroborated these findings and further shown that passive IHC reduces arrhythmias in patients with CAD and produces robust and persistent antihypertensive effects in patients with essential hypertension [7], [34]. The latter effects have recently been convincingly confirmed in hypertensive patients with obstructive sleep apnea: a 3-week program of 15 sessions of passive IHC (2-minute cycles at end-tidal partial pressure of oxygen of 50 mmHg, corresponding to FiO_2_≈0.13) lowered systolic blood pressure by >10 mmHg, from (142.9±8.6) mmHg to (132.0±10.7) mmHg [35]. Endothelial function was investigated in another RCT, including 26 participants aged 60–80 years who received either normoxic (sham) or moderate passive hypoxic stimuli (7 cycles of 5 min hypoxia and 3 min normoxia) for 3 weekly sessions over 8 weeks, to achieve peripheral oxygen saturations of 75%–80%, respectively, after 5 weeks.

The IHC, but not the sham regimen, improved endothelial function, as assessed by flow-mediated dilation of the brachial artery [36]. Noticeably, flow-mediated dilation remained improved 8 weeks after IHC, demonstrating a protracted IHC benefit. The sustained effects of IHC on both NO bioavailability and oxidative stress reduction could augment vascular function [36]. Sustained (up to 4 weeks post IHC) beneficial effects on ambulatory blood pressure in hypertensive patients have been reported independently by several groups, underscoring the improvement in vascular function following IHC [34], [35], [37]. A recent meta-analysis also confirmed the vascular benefits of passive IHC in cardiac patients, indicated by significant reductions in resting heart rate (mean difference: 5.4 beats/min) and in both systolic (mean difference: 13.7 mmHg) and diastolic (mean difference: 7.9 mmHg) blood pressures following IHC, regardless of whether hypoxia-normoxia or hypoxia-hyperoxia cycles were applied [14].

IHC can be combined with exercise through two approaches: 1) exercising during hypoxic exposure (active IHC), or 2) combining passive IHC with separate exercise training as an adjunct intervention. A meta-analysis evaluating active IHC in individuals without CVD found no synergistic benefits of active IHC on the cardiovascular system when compared with normoxic exercise [38]. Those findings suggest that the exercise stimulus is stronger than the additional stimulus imparted by hypoxia, at least in healthy, fit individuals. However, a recent umbrella review of systematic reviews and meta-analyses concluded that active IHC is an effective and adaptable strategy for improving both aerobic and anaerobic performance and enhancing muscle strength and hypertrophy in trained and untrained individuals alike [39].

A more recent approach replaces the normoxic recovery phases between hypoxic exposures with hyperoxic (FiO_2_=0.30–0.40) phases (IHHC). Using passive IHHC alongside exercise as an additional intervention in an RCT led to significant cognitive improvements in geriatric patients [40]. In a recent phase II RCT in individuals with chronic stroke, passive IHC (up to 15 sessions consisting of 15 cycles alternating 60–90 s at FiO_2_=0.08–0.09 and 30–60 s of normoxia) followed by 1 h of high-intensity gait training (in normoxia) yielded superior improvements in locomotor functions vs. high-intensity gait training alone [41]. The impact of added hypoxia on the effectiveness of lifestyle interventions was tested in an RCT involving 20 overweight and obese men undergoing a 4-week program of dietary restriction and exercise training. As compared to the normoxic intervention, active IHC (FiO₂=0.13–0.16) produced more favorable changes in lipid profile and body composition [42]. This study, which lasted between 5 and 7 weeks, proved both easily administered to and well tolerated by geriatric patients (aged 64–92 years). A 6-week passive IHHC program found a trend of beneficial effects on blood pressure, respiratory function, cardiac autonomic activity, and inflammatory markers in older adults [43]. In a controlled clinical pilot project, adding passive IHHC to a multidisciplinary rehabilitation program reduced blood pressure and heart rate, and improved exercise capacity and quality of life in patients with long-COVID-19 [44]. The hyperoxic component (FiO₂=0.30–0.40) accelerates reoxygenation and may therefore reduce the necessary time for IHHC compared to IHC sessions [45]. A direct comparison of IHC and IHHC in prediabetic patients demonstrated the two protocols effected similar reductions in serum glucose, total cholesterol, and low-density lipoproteins [45]. The ongoing scientific and clinical interest in the benefits of hypoxia and IHC is evidenced by the current or planned human studies registered on ClinicalTrials.gov (e.g., intermittent hypoxia therapy in cardiac rehabilitation, ID NCT04034082; effect of training in normobaric hypoxia on cardiac markers, ID NCT06896773; immunometabolic pattern of intermittent hypoxia during ST-segment elevation myocardial infarction, ID NCT05230966).

## 4 Observations from remote ischemic conditioning

Remote ischemic conditioning (RIC) is another conditioning strategy that shares substantial mechanistic and potential clinical overlap with IHC. Concordant with the hormesis principle, RIC aims to limit ischemic injury to an organ such as the heart or brain by triggering the body’s natural protection against tissue injury [46]. RIC involves brief, repetitive cycles of restricting and restoring blood flow to a limb using an inflatable cuff. The effects of RIC in patients undergoing coronary artery bypass grafting or presenting with ST-elevation myocardial infarction were equivocal [47], [48], although promising results were earlier reported in pediatric patients undergoing surgical repair of congenital heart defects [49]. More recently, a large clinical trial found that 67.4% of adult patients randomized to receive RIC within 48 h of symptom onset had excellent neurological function 90 d later, compared to 62.0% of patients receiving usual care (*P*<0.05) [50]. Moreover, RIC recently was shown to significantly improve neurological recovery and reduce disability in patients with mild-to-moderate acute ischemic stroke who did not undergo cerebrovascular recanalization [51]. However, another RCT in 1500 patients with prehospital stroke symptoms for <4 h found that RIC did not improve functional outcome at 90 d vs. sham treatment [52]. Inconsistent RIC outcomes may be partially explained by variations in patient characteristics, standard treatment, and the timing or duration of RIC application, such as 10–14 d in the study by Chen *et al.* [50] vs. 7 d in the study by Blauenfeldt *et al.* [52]. Moreover, the differences among RIC studies may be disease- or organ-specific (e.g., cardiovascular vs. cerebrovascular disease), constitute placebo effects in studies lacking sham intervention groups, or arise from comorbidities and medications, such as propofol and ticagrelor, that may mask or reduce the protective effects of RIC [53], [54].

A recent systematic review concluded that RIC is safe and feasible, and may be effective for promoting recovery from stroke [55]. The most promising regime appears to be chronic RIC, in which treatment commences during the early, acute phase of the illness, and requires that patients maintain the chronic RIC regime for up to 12 months [56]. Stroke is currently one of the most promising areas of application for RIC, as indicated by numerous ongoing or planned clinical trials registered on ClinicalTrials.gov (e.g., blood flow regulation and neuromuscular function post-stroke, ID NCT06027294; safety and efficacy of remote ischemic conditioning in patients with spontaneous intracerebral hemorrhage, ID NCT03484936; safety and efficacy of remote ischemic conditioning in patients with acute ischemic stroke, ID NCT03669653).

Nevertheless, the lack of direct comparisons or combinations of IHC and RIC interventions makes it impossible to compare the respective advantages and disadvantages of each intervention, or the synergistic effects of combining them.

## 5 Core mechanisms

**Fig. 1:**
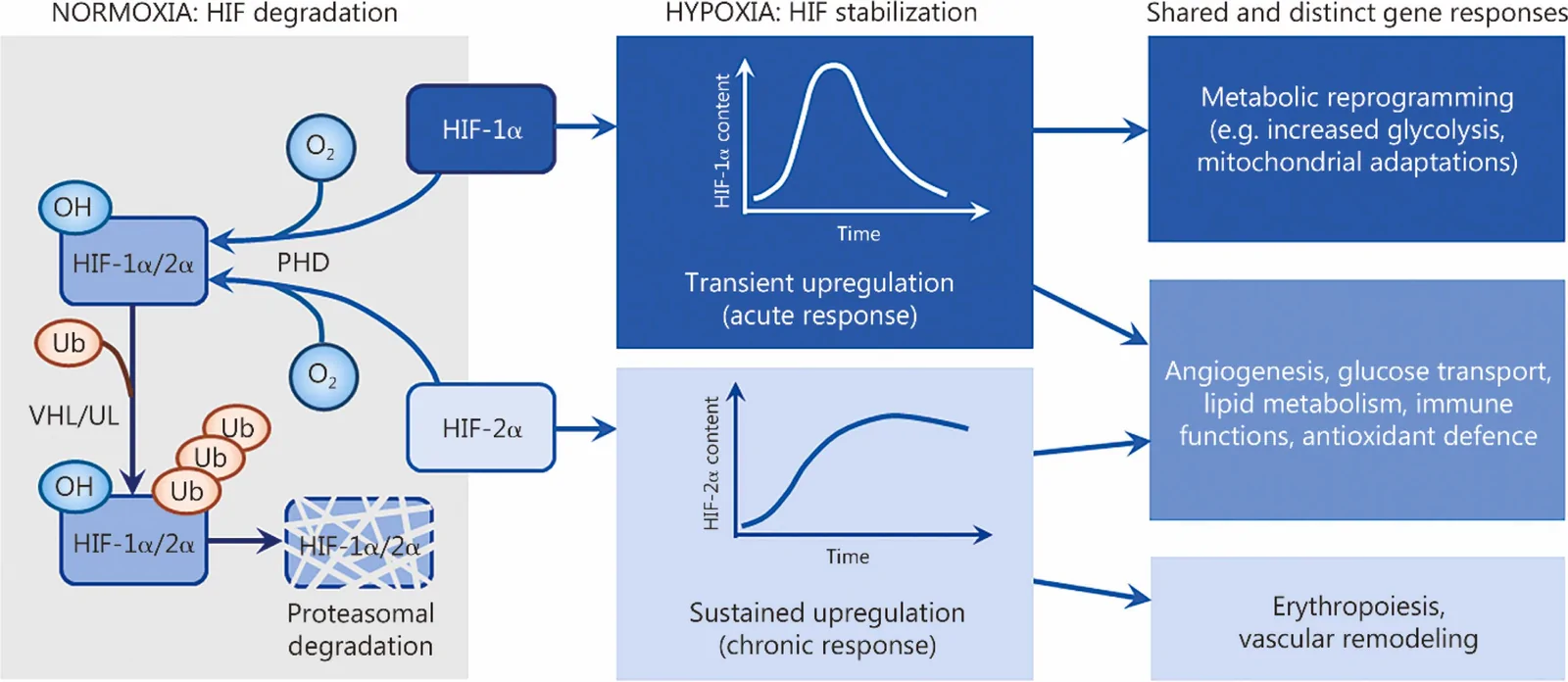
Oxygen-dependent regulation of the central hypoxic response regulators, hypoxia-inducible factors (HIFs). In normoxia, HIF-α subunits are hydroxylated by oxygen-dependent prolyl hydroxylase (PHD), allowing recognition by the von Hippel-Lindau protein (VHL), polyubiquitination (Ub) by ubiquitin ligase (UL), and proteasomal degradation. Low oxygen availability suppresses degradation of HIF-1α and HIF-2α subunits, which migrate to the nucleus, heterodimerize with HIF-1β, and regulate an extensive transcriptomic hypoxia response program. HIF-1α is upregulated rapidly in hypoxia, but after several hours, its levels subside. HIF-2α remains upregulated longer. While HIF-1 primarily regulates metabolic reprogramming, HIF-2 controls erythropoiesis and vascular remodeling. Both HIFs activate angiogenesis and other adaptations to hypoxia responses.

IHC improves cardiovascular function through the hypoxia-induced molecular and cellular cytoprotective signaling cascades eliciting systemic physiological adaptations that limit hypoxic/ischemic injury and endothelial dysfunction. Orchestrating these adaptations are gene programs activated by hypoxia-responsive transcription factors [15], [57], [58], [59]. Reduced cytosolic oxygen concentrations stabilize HIFs, which activate transcription of a portfolio of adaptive genes including those encoding glycolytic enzymes, the pro-angiogenic vascular endothelial growth factor, nitric oxide synthases, and the cytoprotective cytokine erythropoietin (Fig. 1). Moderate hypoxia also generates ROS, which stabilize nuclear factor erythroid 2-related factor 2 (Nrf2) to orchestrate an antioxidant and anti-inflammatory gene program [60]. The protein products of HIF- and Nrf2-activated genes effect metabolic rewiring favoring oxygen-independent glycolytic ATP production, and improve antioxidant defenses, local blood supply, mitochondrial efficiency (e.g., ATP to oxygen ratio), nitric oxide-dependent vasodilation, and cardioprotective bradycardia [7]. Studies mainly in rodents demonstrated several other cardioprotective mediators/effectors, including IHC-induced myocardial heat shock protein 70 [61], inducible nitric oxide synthase [28], [62], and ATP synthase subunit β, mitochondrial aldehyde dehydrogenase, and heat shock protein 27 [63]. Collectively, these mechanisms increase the myocardium’s tolerance to ischemia.

Limited oxygen availability directly affects mitochondrial K^+^ fluxes. IHC activates mitochondrial ATP-sensitive K^+^ (mK_ATP_) channels, which lower mitochondrial oxygen demand and may limit mitochondria-derived ROS overproduction [15]. Concurrently, hypoxia-induced induction of glycolytic enzymes increases the capacity for oxygen-independent ATP synthesis, thereby preserving ATP-dependent electrolyte management and preventing arrhythmias [7]. In addition, IHC provides cardioprotection by augmenting coronary vasodilation in the event of ischemia/reperfusion, an adaptation driven by NF-κB/MuRF1 mediated suppression of degradation of the β_1_ subunits of high-capacity, calcium-activated mitochondrial BK_Ca_ channels [64]. Understanding of the portfolio of molecular mechanisms mediating the cardiovascular benefits of controlled hypoxia is still expanding.

## 6 Safety aspects

In general, IHC programs are well tolerated by both healthy and diseased individuals [14]. A recent systematic review found only two studies that reported mild adverse effects related to IHC in patients with CVD, and there were no significant complications or serious adverse events [14]. Moreover, intraoperative and early postoperative complications did not differ significantly between the treatment arms. Angina episodes occurred in only 6 of 408 IHC sessions (without electrocardiogram abnormalities), and only during the hypoxia exposure of three patients [14]. Recently, 95 adverse events were recorded in a trial, in which 20 participants with Parkinson’s disease underwent different IHC protocols [65]. However, those events were not dependent on hypoxia dose and showed similar incidences in the hypoxia and normoxic placebo conditions. Nevertheless, careful patient selection is a fundamental prerequisite of clinical IHC application. Particular caution is advised in the presence of risk factors or medical conditions for which the past clinical experience of IHC is lacking. At least in facilities that are not specialized centers, severe and acute illnesses should generally be considered contraindications for IHC practice, including unstable cardiovascular, pulmonary, and neurological conditions, malignancies (due to the risk of cancer progression and metastasis), and the acute phases of heart attacks, strokes, or severe infections. It is strongly recommended that medical intervention with IHC be overseen by appropriately trained and equipped professionals.

## 7 What remains to be done?

### 7.1 Distinguishing therapeutic from pathological hypoxia

**Fig. 2:**
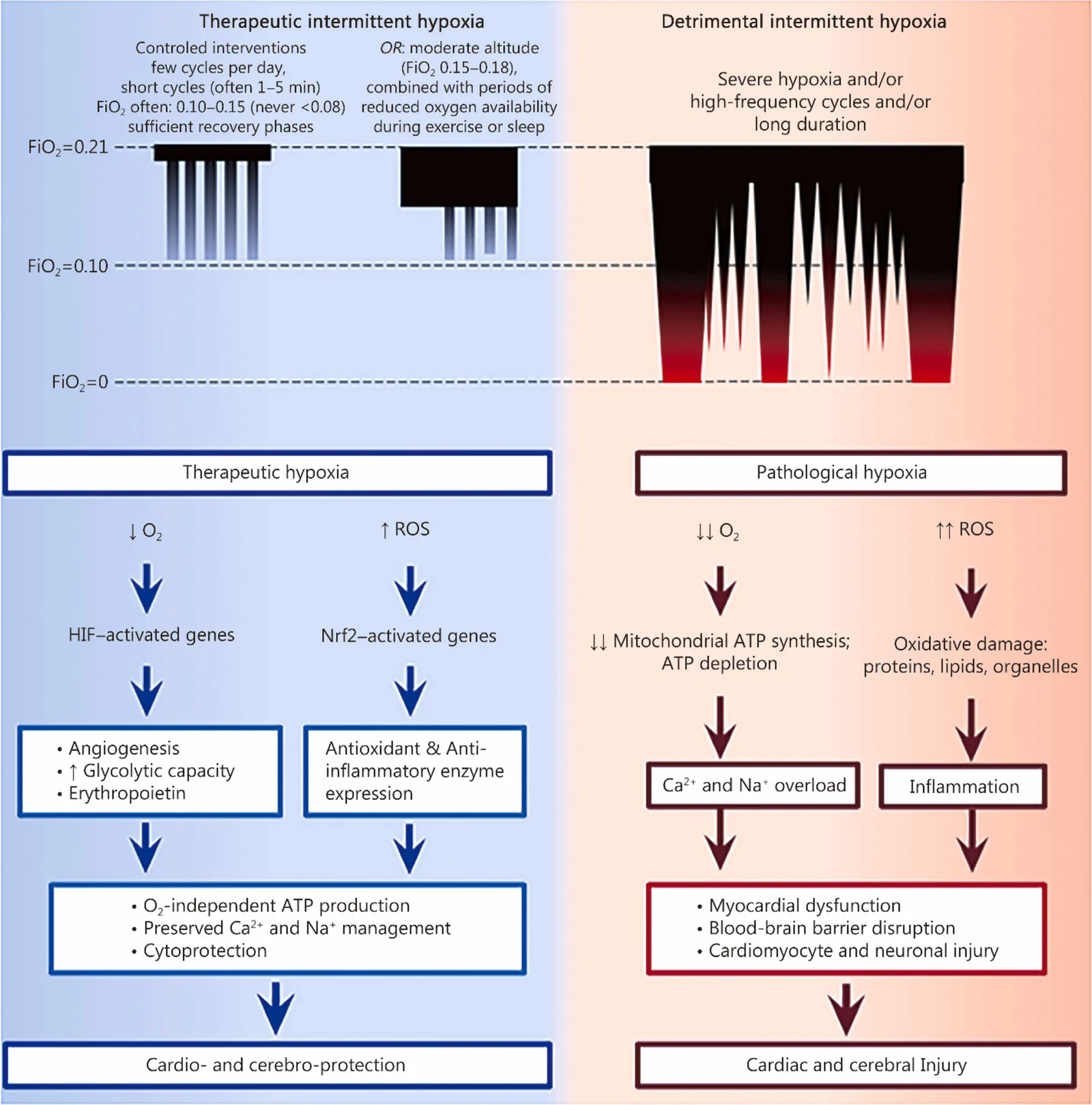
Divergent effects and underlying mechanisms of therapeutic vs. pathological hypoxia on heart and brain. Intermittent hypoxia protocols conferring cardio- and cerebro-protection consist of a limited number of brief (1–5 min) cycles of moderate [fraction of inspired O_2_ (FiO_2_) typically 0.10–0.15, never <0.08] hypoxia-reoxygenation with sufficient recovery phases, superimposed on normoxia or mild hyperoxia (FiO_2_=0.30), which activate hypoxia- and reactive oxygen species (ROS)-responsive gene expression, to maintain ATP production and manage ROS overproduction during severe hypoxia-ischemia/reperfusion (left). Intense, protracted, or high-frequency episodic hypoxia, e.g., that imposed by vascular occlusion or sleep apnea, compromises ATP-dependent ion transport and inflicts cytotoxic oxidative stress and inflammation (right). HIF. Hypoxia-inducible factor; Nrf2. Nuclear factor erythroid 2-related factor 2.

It is paramount to distinguish therapeutic IHC from uncontrolled, severe, pathologic hypoxia (e.g., that occurring during sleep apnea) and from pathological hypoxic conditions at the systemic (e.g., consequences of respiratory or cardiovascular diseases) and tissue (e.g., tumor hypoxia, inflammatory hypoxia) levels. An overview of the distinct mechanisms potentially underlying pathological and therapeutic hypoxia is presented in Fig. 2. Because the delimitation of pathological versus therapeutic forms of hypoxia is poorly defined, IHC is not yet a widely accepted complementary clinical strategy despite the unequivocal cardiovascular benefits and practical advantages of well-calibrated IHC as a safe, simple, non-pharmacological tool.

### 7.2 Defining the IHC dose

Although the safety of common IHC protocols is well established, residual concerns are understandable regarding the IHC-parameters for optimal therapeutic benefit, including FiO_2_ and durations of the hypoxic and normoxic/hyperoxic recovery intervals, individual control (e.g., via feedback mechanisms), the number of hypoxia-normoxia cycles per session, the progression of cycle duration and number, sufficient recovery periods between intervals and sessions, and the optimal program duration (days or weeks). These IHC parameters remain inadequately defined, and clear professional guidelines for IHC intervention are currently lacking.

### 7.3 Determining the persistence of effects and potential booster protocols

Although certain benefits, such as reductions in blood pressure [35] and serum glucose and lipids [45], have been found to persist one month or longer following IHC programs, it is unclear if all IHC benefits are similarly protracted. As with the hypoxic dose, the optimal timeframe for re-induction of IHC after its initial benefits subside is not yet established and likely varies among individual patients and clinical conditions. The impacts of medical history, comorbidities, medications, and genetics on IHC induction, persistence, and reinduction are unclear and merit clinical investigation.

### 7.4 Positioning IHC relative to RIC

**Fig. 3:**
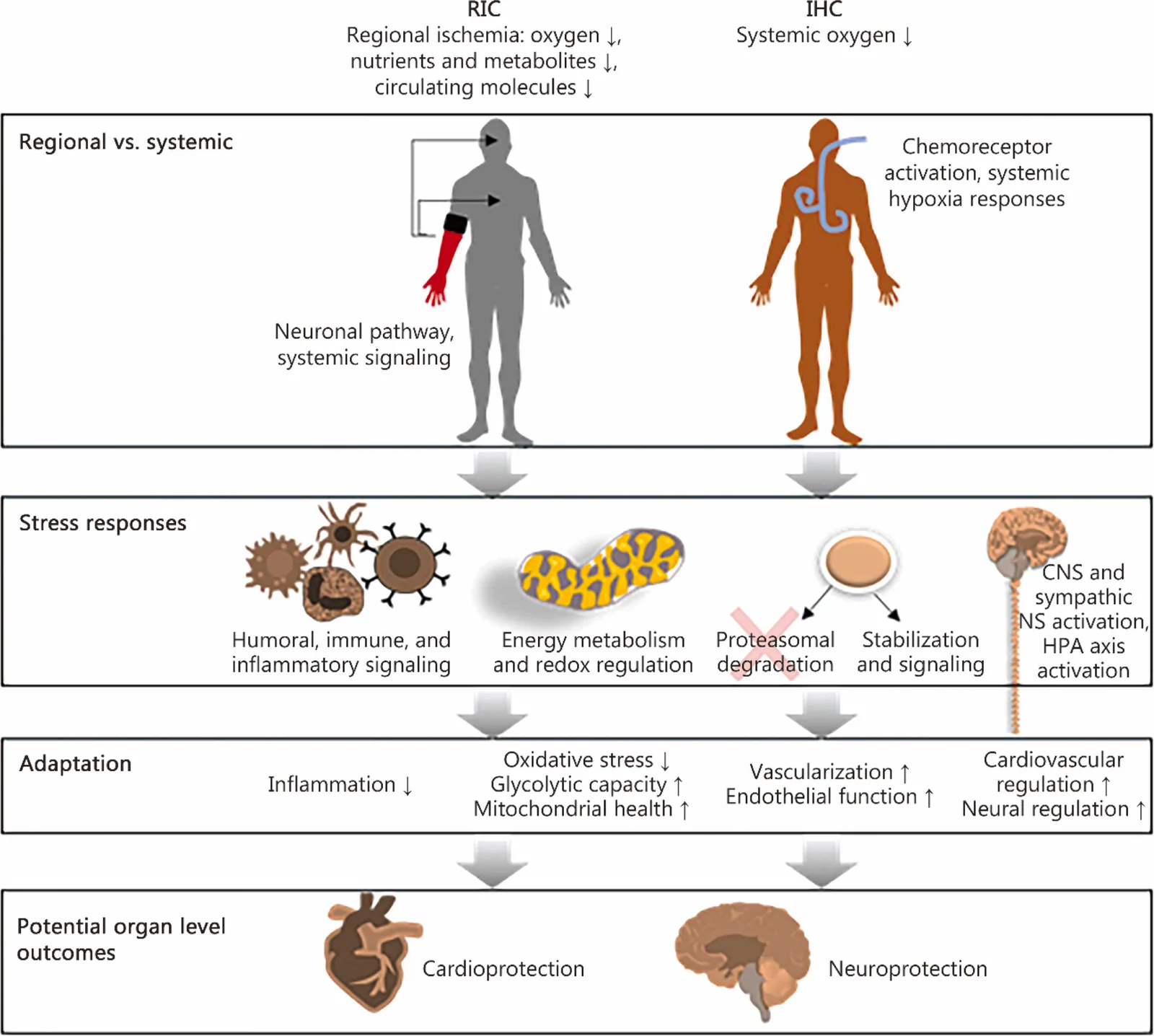
Induction of complementary stress responses by remote ischemic conditioning (RIC) and intermittent hypoxia conditioning (IHC) leads to overlapping mechanisms potentially leading to protective outcomes. Since optimal RIC and IHC protocols are as yet unestablished, studies directly comparing the two approaches are lacking, and claims of superiority of one approach over the other are speculative. However, protocols exist for both RIC and IHC that are widely considered safe. A major challenge will be tailoring those protocols to the needs of specific medical conditions and individuals. CNS. Central nervous system; NS. Nervous system; HPA. Hypothalamic-pitutary-adrenal.

It will also be important to disentangle differences related to mechanisms and clinical applications between IHC and RIC modalities. IHC and RIC share similar mechanistic pathways, including those impacting HIF-signaling, mitochondrial function, redox regulation and inflammation [11]. Commonly used IHC and RIC protocols are considered safe and potentially therapeutically effective in cardiovascular and neurological diseases [14], [55]. However, beneficial effects are induced differently: in RIC, regional ischemia is commonly caused by limb cuffs, while IHC utilizes reduced inspiratory oxygen [11]. Thus, for effects on the heart and brain, RIC probably primarily relies on circulating humoral and other signaling molecules released from the cuffed limb in response to ischemic stress signaling (including HIF-pathway activation). In contrast, IHC causes brief, systemic reductions of oxygen saturation, sensed by peripheral and central chemoreceptors and triggering whole-body cellular HIF-signaling in cells affected by reduced oxygen availability [60]. Therefore, the two methods place different emphasis on the importance of the involved signaling pathways. Direct comparisons of the efficacy of RIC and IHC are lacking, but most likely, the suitability of each approach depends on the medical condition. Similar to IHC, optimal dosages for RIC in specific conditions remain to be established, which impedes direct comparison of the methods and possibly accounts for the inconsistency of results, such as of clinical trials on RIC in stroke [52]. A putative comparison of RIC and IHC is presented in Fig. 3. Unfortunately, we are not aware of any studies that offer suitable comparisons of the effectiveness of IHC and RIC interventions, despite the substantial overlap of their mechanisms. However, such studies could help define the areas of application for both interventions and provide insights into possible synergistic effects.

## 8 Conclusions

There is mounting clinical evidence supporting the beneficial preventive and therapeutic effects of IHC on vascular function, the heart, and, although less conclusively demonstrated, on the brain. IHC is likely to be particularly successful when combined with exercise interventions, especially in patients with low cardiorespiratory fitness. Feasibility of IHC administration is high, and safety concerns are low. However, the clinical application of IHC also presents challenges, including the need for standardized protocols for different conditions (e.g., different diseases and disease states, medical histories and medication profiles), evaluating the long-term effects of IHC, and determining the time intervals at which IHC re-administration is needed. Ultimately, personalized IHC treatment strategies could be refined by integrating modern medical technologies, such as artificial intelligence. Comparisons that suitably differentiate the effects of IHC and RIC interventions would help to identify the most promising areas of application for both strategies.

## Abbreviations

ATP: Adenosine triphosphate

BKCa: Large-conductance calcium-activated potassium channel

CAD: Coronary artery disease

CVD: Cardiovascular disease

FiO_2_: Fraction of inspired O_2_

HIF: Hypoxia-inducible factor

IHC: Intermittent hypoxia conditioning

IHE: Intermittent hypoxic exposure

IHHC: Intermittent hypoxia-hyperoxia conditioning

IHHT: Intermittent hypoxia-hyperoxia training

IHT: Intermittent hypoxia training

mKATP: Mitochondrial ATP-sensitive potassium channel

MuRF1: Muscle RING-finger protein 1

NF-κB: Nuclear factor κB

Nrf2: Nuclear factor erythroid 2-related factor 2

O_2_: Oxygen

RCT: Randomized controlled trial

RIC: Remote ischemic conditioning

ROS: Reactive oxygen species

## Ethics approval and consent to participate

Not applicable.

## Funding

Not applicable.

## Data Availability

Not applicable.
